# Commentary: From ‘sense of number’ to ‘sense of magnitude’ – The role of continuous magnitudes in numerical cognition

**DOI:** 10.3389/fpsyg.2016.02032

**Published:** 2017-01-04

**Authors:** Peter Kramer, Paola Bressan

**Affiliations:** Department of General Psychology, University of PaduaPadova, Italy

**Keywords:** sense of number, sense of magnitude, numerosity estimation, occupancy, statistical learning

Unlike abstract ones in mathematics, concrete sets of elements in the real world have continuous physical properties, such as overall area and density. The dominant view has it that humans can estimate the discrete numerosities of such sets independently of the co-varying continuous magnitudes; i.e., that humans have a “sense of number.” It has indeed been claimed that various animals, ranging from monkeys to tiny fish, have this sense too. A recent paper by Leibovich et al. (2016) questions all of this (see also Morgan et al., 2014; Gebuis et al., 2016) and argues convincingly that numerosity estimation is not independent from continuous magnitudes but relies on them; that we have not a “sense of number” but a “sense of magnitude.”

Yet the authors fail to cite a classic article that made the very same argument 25 years ago, and—unlike Leibovich et al.—supported it with a quantitative model (Allik and Tuulmets, 1991). Although neither density, nor overall area, nor any other single continuous magnitude can provide reliable information about numerosity, Leibovich et al. imply that all of them together can; they suggest that “statistical learning” will take care of extracting this information and turn numerosity estimates out of it. How statistical learning achieves this feat and whether the resulting numerosity estimates will fit observed ones remains, unfortunately, unclear. Allik and Tuulmets's alternative “occupancy” model has its limits (e.g., Kramer et al., 2011; Bertamini et al., 2016) but it is specific, it is quantitative, and it predicts observed numerosity estimation surprisingly well with just a single free parameter.

To understand the occupancy model, consider a set of identical dots and imagine that each of them is covered with a larger disk, as in Figure 1. The model posits that the total area occupied by the disks (*occupancy*) will be linearly related to the estimated numerosity of the dots. As long as dot densities remain relatively low (Durgin, 1995), the model accounts for nearly 90% of the variance in human data (Allik and Tuulmets, 1991). The closer together the dots are and thus the more the disks overlap, the smaller occupancy is and thus the smaller the estimated numerosity of the dots is predicted to be—as indeed observed (e.g., DeWind et al., 2015). Notably, because large numerosities tend to be dense, the occupancy model predicts they will tend to be underestimated. This prediction has been corroborated repeatedly too (e.g., Izard and Dehaene, 2008). How Leibovich et al.'s statistical learning could make the same prediction is hard to tell.

**Figure 1:**
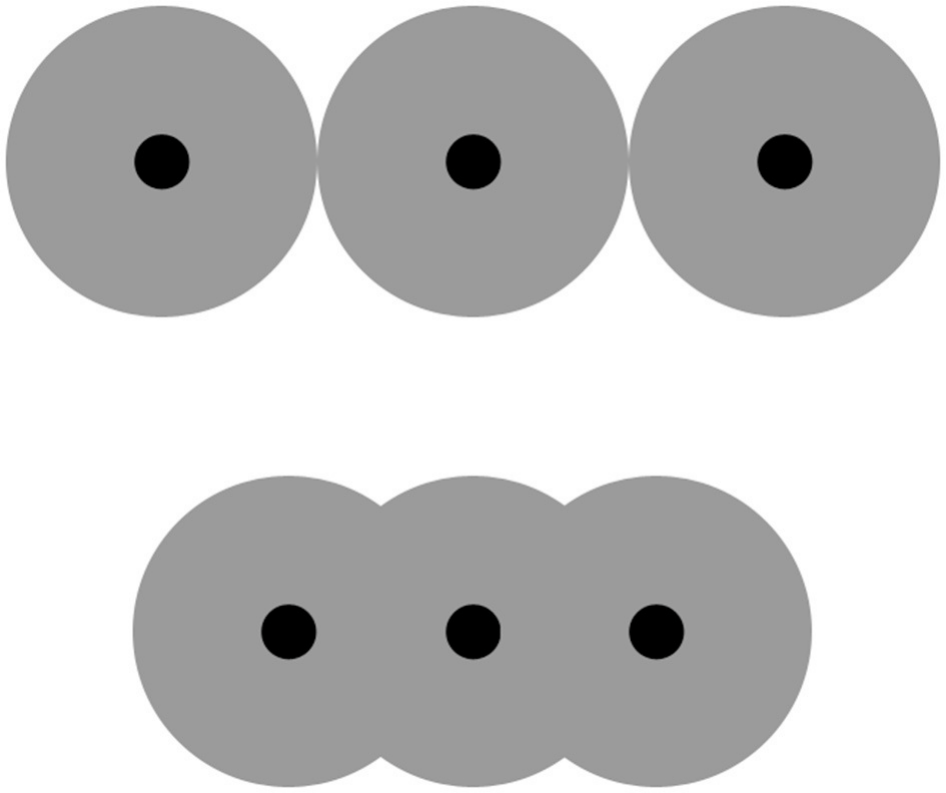
**Effect of item spacing on occupancy**. The “occupancy” of a dot may be represented as a larger, concentric virtual disk that covers it, shown here in gray. (The radius of the disk is a free parameter that, during model fitting, Allik and Tuulmets estimated to be 0.33° of visual angle.) The three black dots in the bottom row are closer together than those in the top row. As a result, the virtual disks in the bottom row overlap, making for a smaller occupancy (total gray area) and therefore a smaller predicted numerosity estimate. For illustration purposes only a few dots are shown; note, however, that numerosity estimation concerns numerosities outside the “subitizing” range of one to four items.

The occupancy model—even in Durgin's (1995) variant—is too simple to work under all conditions (Kramer et al., 2011). Still, if the degree of overlap of the virtual disks covering the dots is interpreted as an inverse measure of the mutual discriminability of the dots, the model makes an intuitive point. The point is that the less discriminable from one another items are, the lower their estimated numerosity ought to be. If so, the occupancy model could be expanded and improved (Kramer et al., 2011) by taking into account anything that affects the items' (1) discriminability (their distance from one another and from fixation, for example), (2) retention across saccades (if presentation times allow eye movements), and (3) retention in memory (if the stimulus is presented or inspected sequentially or compared to another one).

Although people are under the impression they are blessed with a high-resolution image of the world around them, careful experimentation has revealed that this is largely an illusion (e.g., Durgin, 1995). In numerosity-estimation experiments the stimuli tend to be randomly placed items, usually dots, and some of them end up clumped together. These items may be impossible to discriminate from one another, even when they can all be perceptually detected. This *crowding effect* (Whitney and Levi, 2011) is a serious concern, because the opportunities for crowding necessarily grow with numerosity and have been shown to affect its estimation (Valsecchi et al., 2013; Anobile et al., 2015).

So far, studies that claim that numerosity estimation does not fully rely on continuous magnitudes, or that it is more cognitive than perceptual in nature, have not been very specific about how the perceptual system extracts numerosity from the stimulus (see also Morgan et al., 2014). These studies have considered a wide range of perceptual factors such as density, overall area, and item size. None of these factors, however, has ever been claimed to be crucial in numerosity estimation. The one factor that has for as long as 25 years (i.e., occupancy) is instead consistently ignored, including now by Leibovich et al. (2016). Izard and Dehaene (2008), for example, do cite Allik and Tuulmets (1991) and control for various factors including “occupied area,” but they use the term to mean overall area (which nobody considers critical to numerosity estimation) and not occupancy (which Allik and Tuulmets themselves consider critical). Izard and Dehaene dismiss occupancy as “a complex combination” of two parameters (density and occupied area). Occupancy does not strike us as complex, but even if it were, the fact that it reflects well-established perceptual discriminability limitations that have been demonstrated to affect numerosity estimation—rather than hypothetical cognitive ones that continue to be debated—should give the occupancy model an edge over any “sense of number” model. In defense of their own “sense of magnitude” model, which Leibovich et al. fail to cite too, Morgan et al. (2014) make a similar point.

The occupancy model deserves at the very least discussion—if not revival and development. It has the potential to challenge not only “sense of number” models but also Leibovich et al.'s statistical-learning alternative to them. Unlike the latter, the occupancy model is a “sense of magnitude” model that does not require learning, is deterministic, quantitative, perception- rather than cognition-based, and not inconsistent with the idea that numerosity estimation might be innate and so simple that even fish could do it.

## Author contributions

PK wrote the first draft of the manuscript; both authors discussed it, critically revised it, and agreed on the final version.

### Conflict of interest statement

The authors declare that the research was conducted in the absence of any commercial or financial relationships that could be construed as a potential conflict of interest.

## References

[B1] Allik J. Tuulmets T. (1991). Occupancy model of perceived numerosity. Percept. Psychophys. 49, 303–314. 10.3758/BF03205986 2030927

[B2] Anobile G. Turi M. Cicchini G. M. Burr D. C. (2015). Mechanisms for perception of numerosity or texture-density are governed by crowding-like effects. J. Vis. 15, 1–12. 10.1167/15.5.4 26067522 PMC4909146

[B3] Bertamini M. Zito M. Scott-Samuel N. E. Hulleman J. (2016). Spatial clustering and its effect on perceived clustering, numerosity, and dispersion. Atten. Percept. Psychophys. 78, 1460–1471. 10.3758/s13414-016-1100-0 27142523 PMC4914534

[B4] DeWind N. K. Adams G. K. Platt M. L. Brannon E. M. (2015). Modeling the approximate number system to quantify the contribution of visual stimulus features. Cognition 142, 247–265. 10.1016/j.cognition.2015.05.016 26056747 PMC4831213

[B5] Durgin F. H. (1995). Texture density adaptation and the perceived numerosity and distribution of texture. J. Exp. Psychol. Hum. Percept. Perform. 21, 149–169.

[B6] Gebuis T. Cohen Kadosh R. Gevers W. (2016). Sensory-integration system rather than approximate number system underlies numerosity processing: a critical review. Acta Psychol. 171, 17–35. 10.1016/j.actpsy.2016.09.003 27640140

[B7] Izard V. Dehaene S. (2008). Calibrating the mental number line. Cognition 106, 1221–1247. 10.1016/j.cognition.2007.06.004 17678639

[B8] Kramer P. Di Bono M. G. Zorzi M. (2011). Numerosity estimation in visual stimuli in the absence of luminance-based cues. PLoS ONE 6:e17378. 10.1371/journal.pone.0017378 21387017 PMC3046164

[B9] Leibovich T. Katzin N. Harel M. Henik A. (2016). From ‘sense of number’ to ‘sense of magnitude’ - the role of continuous magnitudes in numerical cognition. Behav. Brain. Sci. [Epub ahead of print]. 10.1017/S0140525X16000960. 27530053

[B10] Morgan M. J. Raphael S. Tibber M. S. Dakin S. C. (2014). A texture-processing model of the ‘visual sense of number’. Proc. Biol. Sci. 281:20141137. 10.1098/rspb.2014.1137 25030988 PMC4123707

[B11] Valsecchi M. Toscani M. Gegenfurtner K. R. (2013). Perceived numerosity is reduced in peripheral vision. J. Vis. 13, 1–16. 10.1167/13.13.7 24198398

[B12] Whitney D. Levi D. M. (2011). Visual crowding: a fundamental limit on conscious perception and object recognition. Trends Cogn. Sci. 15, 160–168. 10.1016/j.tics.2011.02.005 21420894 PMC3070834

